# 

**Published:** 1916-01

**Authors:** 


### OUR NEW SUBSCRIBERS.

Dr. W. G. Bryan ----------Ariz.
Dr. J. M. King -----------Ark.
Dr. Chas. L. Smith--------Texas
Dr. H. S. Woodward------Texas
Dr. H. G. Camper ------West Va.
Dr. K. M. Jarrell------West Va.
Dr. O. G. Doughtery --------Ky.
Dr. H. McLeod -----------Ala.
Dr. Wm. Mann ----------N. C.
Dr. C. F. Hawley ---------Ariz.
Dr. B. F. Clutter --------Texas
Dr. H. A. Reese -----------Ariz.
Dr. W. T. Anderson ------Texas
Dr. W. T. Gabbert --------Ark.
Dr. Geo. A. Bridge--------Ariz.
Dr. Paul C. Starkey --West Va.
Dr. G. C. Robertson ---West Va.
Dr. F. F. Fadeley-----New Mex.
Dr. J. D. Jackson ---------Ky.
Dr. R. Dillard -----------N. C.
Dr. C. N. McGaffey ------Texas
Dr. Thos. L. Nutter---West Va.
Dr. G. T. Abernathey -----Tenn.
Dr. J. H. oRwsey ------West Va.
Dr. P. H. Swann ------West Va.
Dr. G. T. Divers -----------Va.
Dr. J. H. Green ----------Tenn.
Dr. R. F. Herndon -------Texas
Dr. E. A. Drum ------------Va.
Dr. Chas. Blickensderfer---Okla.
Dr. C. G. Meritt-----------Texas
Dr. J. T. Altman ----------Ark.
Dr. T. C. Savage ----------Ala.
Dr. H. B. Wilkinson -------Ala.

Dr. A. P. Webb ____________Ala.
Dr. B. W. Black __________Texas
Dr. W. R. Bashiell _________Texas
Dr. C. W. Bartlett __________Fla.
Drs. Crume & Killough____Texas
Dr. A. F. Lumpkin _______Texas
Dr. L. T. Shirley __________S. C.
Dr. D. Y. Moore _____________Ga.
Dr. J. E. Fisher __________Tenn.
Dr. R. L. Ray _____________Tenn.
Dr. E. L. Stalworth _________Ala.
Dr. Buchan & Buchan ______Ark.
Dr. W. L. Parchman _______Ark.
Dr. W. D. Chapelle, Jr._____S. C.
Dr. W. C. Dunnaway ______Ark.
Dr. G. L. Boykin __________S. C.
Dr. W. W. Whitington______N. C.
Dr. J. H. Murray ____________La.
Dr. Wm. L. Thorne ________S. C.
Dr. W. W. Crawford ______Miss.
Dr. E. L. Cox ____________N. C.
Dr. B. H. Henry ___________S. C.
Dr. L. B. Bland __________Texas
Dr J. A. Lightfoot ______Texas
Dr. John S. Turnne _______Texas
Dr. E. O. Boggs ___________Texas
Dr. J. B. Chapman ________Texas
Dr. A. G. Payne ___________Miss.
Dr. W. B. Chamberlin ________La.
Dr. M. J. Lingo ____________Fla.
Dr. M. Dennis ____________Texas
Dr. Jos. D. Collins ___________Va.
Dr. T. C. Thorington ________Ala.
Dr. W. F. Rose _____________Ark.
Dr. McCarthy & McCarthy__Tex.
Dr. S. L. Wolferman ______Ark.
Dr. J. M. Winchester __New Mex.
Dr. H. C. Riley ____________Ala.
Dr. H. H. Burke ____________Va.
Dr. J. W. Duke ___________Okla.
Dr. L. E. Andrews ________Okla.
Dr. Gillis & Fair _________Okla.
Dr. C. F. Ellis _____________Ark.
Dr. E. W. Horton _____West Va.
Dr. E. W. Baron __________S. C.
Dr. S. G. Von Almen__New Mex.
Dr. S. B. Moore ____________S. C.
Dr. J. T. Coggeshall ________S. C.
Dr. H. T. Heflin ___________Ala.
Dr. W. H. Pickels _________Texas
Dr. D. L. Eastland ________Texas
Dr. Robt. P. Morehead ____N. C.
Dr. Jas. A. McFerrin ______Tenn.
Dr. T. Adair _____________W. Va.
Dr. K. Bradford ____________Ala.
Dr. A. M. Williams _________Ala.
Dr. N. J. Kennedy __________S. C.
Dr. W. P. Rowan_________W. Va.
Drs. McKinney & Burns______Ark.
Dr. R. B. Nutter ______West Va.
Dr. W. H. Fisher ___________Md.
Dr. J. L. Barker ____________Ky.
Dr. E. A. Abernathy ________La.
Dr. Wm. R. Martin _________Va.
Dr. W. G. White __________S. C.
Dr. H. D. Hively ______West Va.
Dr. K. P. B. Bonner________N. C.
Dr. A. E. Evans ____________Va.
Dr. G. D. Huddleston ______Ark.
Dr. W. L. Walker ___________Ky.
Dr. R. S. Carroll __________N. C.
Drs. Blaylock & Holland___Okla.
Dr. F. W. Francis _______Texas
Dr. Alise Moses _____________Ga.
Dr. B. I. Lewis _____________Ala.
Dr. O. A. Ryder ____________Va.
Dr. L. E. Clark __________Texas
Dr. L. R. Talley _________Texas
Dr. L. H. Talley _________Texas
Dr. C. W. Archer _________Texas
Dr. W. G. Harris _________Texas
Dr. L. I. Collier __________Texas
Dr. D. M. Moore __________S. C.
Dr. J. G. Stuart ____________S. C.
Dr. Wm. G. Sommerville__Tenn.
Dr. F. H. Walke ____________La.
Dr. L. M. Stokes __________S. C.
Dr. J. L. Hobbs ______New Mex.

Dr. J. H. Williams _______Tenn.
Dr. J. A. Amon ___________Ky.
Dr. J. S. Hume ___________Okla.
Dr. I. N. McCollum _______Ark.
Dr. R. M. McDaniels_____Texas
Dr. M. J. Payne ___________Va.
Dr. Wm. Howe _______New Mex.
Dr. J. C. A. Guest _______Texas
Dr. L. H. Graham ________Texas
Dr. C. V. Unsworth _________La.
Dr. J. P. McAnulty ______Texas
Dr. W. P. Peters __________Va.
Dr. W. M. Palmer _________Md
Dr. T. A. Duggan ___________La.
Dr. P. M. Judy ___________S. C.
Dr. A. B. Kalbaugh ________Md.
Dr. C. F. Thompson ______Tenn.
Dr. W. F. Rainey _________N. C.
Dr. I. H. Moore __________Texas
Dr. E. A. Allin __________Texas
Dr. J. W. Harned __________Ky.
Dr. G .M. Truluck ________S. C.
Dr. H. G. Tonkin ______West Va.
Dr. J. J. Henderson____West Va.
Dr. R. L. Foster ___________Ky.
Dr. Earl H. Hunt __________Ark.
Dr. Chas. H. Cargile _______Ark.
Dr. L. Y. King ___________S. C.
Dr. Albert Rockuet _________La.
Dr. J. P. Wales __________Del.
Dr. H. P. Taylor ___________Ky.
Dr. F. E. Shine __________Ariz.
Dr. W. H. Wallingford __W. Va.
Dr. H. D. Allen ____________Ga.
Dr. T. A. Walker __________La.
Dr. J. M. Pemberton ______Okla.
Dr. A. C. Waldrep _________Ala.
Dr. W. H. Abington _______Ark.
Dr. Steve Hawes __________N. C.
Dr. R. L. Hugins __________Va.
Dr. P. E. Johnson _________Ark.
Dr. Lee Yates ___________Texas
Dr. C. R. Enslow _______W. Va.
Dr. R. K. McClanahan__N. Mex.
Dr. Jas. Ofl Greenlaw ___N. Mex.
Dr. T. E. Aashinhurst ____Okla.
Dr. T. J. Caldwell ________Texas
Dr. C. C. Hill _____________S. C.
Dr. J. E. Cannaday _____W. Va.
Dr. T. T. Carlisle _________Dell.
Dr. W. A. Stevensn _______Miss.
Dr. T. J. Woolridge _________Va.
Dr. T. W. Perkins __________Ky.
Dr. R. P. Bell _____________Va.
Dr. J. W. Allen____________Miss.
Dr. Julian Adair __________Del.
Dr. F. Young ______________Ala.
Dr. E. H. Ball ________West Va.
Dr. A. S. Peck ___________S. C.
Dr. W. H. Mock ___________Ark.
Dr. John M. Stanley ______Miss.
Dr. G. A. Schaub ________Texas
Dr. J. P. Davison _____West Va.
Dr. J. M. Elderdice _________Md.
Dr. S. L. Terrell _________Texas
Dr. H. E. Morris ________Texas
Dr. S. L. Burton _____New Mex.

